# Wavelets based physics informed neural networks to solve non-linear differential equations

**DOI:** 10.1038/s41598-023-29806-3

**Published:** 2023-02-18

**Authors:** Ziya Uddin, Sai Ganga, Rishi Asthana, Wubshet Ibrahim

**Affiliations:** 1 SoET, BML Munjal University, Gurugram, Haryana 122413 India; 2 Department of Mathematics, Ambo University, Ambo, Ethiopia

**Keywords:** Mechanical engineering, Applied mathematics, Computational science, Computer science

## Abstract

In this study, the applicability of physics informed neural networks using wavelets as an activation function is discussed to solve non-linear differential equations. One of the prominent equations arising in fluid dynamics namely Blasius viscous flow problem is solved. A linear coupled differential equation, a non-linear coupled differential equation, and partial differential equations are also solved in order to demonstrate the method’s versatility. As the neural network’s optimum design is important and is problem-specific, the influence of some of the key factors on the model’s accuracy is also investigated. To confirm the approach’s efficacy, the outcomes of the suggested method were compared with those of the existing approaches. The suggested method was observed to be both efficient and accurate.

## Introduction

Physics informed neural networks (PINNs), a type of machine learning approach, can be used to find the solution of differential equations by including all of the physics into the loss function and building a neural network that approximates the solution. In PINN, the neural network is optimized in such a way that the loss function is taken as a residual of the governing differential equation, boundary conditions, and initial conditions. The fundamental idea of PINN is that the neural network approximates the solution of a differential equation and satisfies any given constraints such that the loss function is minimized. A few of the earliest examples of using artificial neural networks to determine the solution of differential equations are in the work of Dissanayake et al.^1^ and I.E. Lagaris et al.^2^. The differential equation is presumed to be satisfied by a trial solution in the approach suggested by Lagaris et al.. The trial solution is defined as the sum of two terms, where one term satisfies the initial or boundary conditions and the other term is a neural network approximation that has no impact on the initial or boundary conditions. However, finding the trial solution could be challenging for more complex problems. Later, Raissi et al.^3^ suggested Physics Informed Neural Networks (PINN), the approach for solving differential equations without a trial function. In the past few years, this method has been extensively applied to solve different differential equations. The research on the optimal PINN architecture is still ongoing, and there has been a recent surge in work on a wide range of problems. Some PINN research concentrated on the building and training of neural networks. Effects of network architecture on solving different problems have also been observed^4–8^. Researchers have also looked at how the size of the neural network affects estimation accuracy^9^. The performance of PINN training is also significantly influenced by the activation function. Adaptive activation functions have been introduced, which are proved to improve convergence in neural networks^10^. A comprehensive survey of the various activation functions that have been employed over time can be found in^11^. This work also compares the effectiveness of several activation functions on various test cases. Many investigations have been made into how the number of neurons, hidden layers, and activation functions affect the PINN’s quality of approximation^12^. Different optimization methods and their improvements have also been studied^9^. Other than the modifications in hyper parameters, there have also been several works on developing various kinds of PINNs, like XPINN^13^, VPINN^14^, hp-PINN^15^, GPINN^16^ that integrate techniques from some traditional methods. Different possibilities for hybridizing PINN have also been discussed^17^. Other works address the impact of adding more information to the loss function, which leads to improved performance^18^, as well as the impact of assigning dynamic weights to the loss function^19^. Because of its high flexibility and expressive ability, PINN has been used to solve various problems^20^. There have also been few works on the theoretical side of PINN^21–23^. Because of the advancement of deep learning in methodology, algorithms, and theory, the study of PINN is still an important area of research^12^. An extensive review of PINN can be found in^24,25^. Extreme learning machines, a type of machine learning algorithm used to solve differential equations, have been the focus of recent research^26,27^. The approach takes into account key properties that set it apart from conventional gradient-based methods and these techniques address some of the PINN’s limitations. The description of the technique can be found in^28^. Numerous variations of this technique that combine PINN and ELM have been investigated in^29,30^. The Extreme theory of functional connection^31^, a novel technique that was recently established, combines PINN and the theory of functional connections approach. For several problems, it has been seen that this method provides good accuracy and computational efficiency. Mortari^32^, Leake et al.^33^ and Leake and Mortari^34^ provided further details about the new methodology, the theory of functional connections.

Finding the solution to ill-posed, inverse, and high-dimensional problems that arise in diverse applications through traditional approaches is comparatively difficult. PINN has advantages over classical methods in such scenarios. PINN is a mesh-free method, and for approximating the solution, one needs to determine only the unknown parameters of the approximate neural network. Hence the task of generating meshes in higher dimensions is shifted to the training of neural networks, making PINN a flexible approach^25^. The solutions that are obtained from PINN are differentiable and can therefore be used in later calculations. Even though there has been a lot of work done, there is still scope for the study of PINN in different applications. Using wavelet as an activation function in PINN is observed to have certain advantages. Because of the nice properties of wavelets, neural networks with wavelet activation functions have better generalization capability. Zainuddin et al.^35^ have used wavelet activation function for predicting the time-series pollution data and observed that the learning speed of neural networks using wavelets as an activation function is relatively higher.

Using a wavelet, one can represent a given function in several scale components^36^. The wavelet function ($\psi$) and the scaling function ($\phi$) are the two functions that define wavelets. Because of the excellent properties of wavelets, many researchers have shown a strong interest in numerical analysis using wavelet theory. Wavelets can represent a given function with a lower number of coefficients and a faster algorithm. A few other properties include space and frequency localization. The important studies in wavelet theory are those given by Stromberg^37^, Grossmann and Morlet^38^, and Meyer^39^. Mallat^40^ and Daubechies^41^ also made significant contributions to the concept. There are several wavelets discussed in the literature with distinct properties. Three different wavelets are considered in this study namely the Morlet wavelet function, the Mexican hat wavelet function, and the Gaussian wavelet function.

In this study, PINN using wavelet as an activation function is applied to solve five problems: firstly the Blasius equation (a nonlinear differential equation defined on an unbounded domain), a linear and non-linear coupled equation, and then the Burger’s equation for two different cases (nonlinear partial differential equation).

Blasius equation is one of the prominent equations arising in fluid dynamics. It governs the boundary layer which appears on a semi-infinite flat plate with a steady two-dimensional laminar flow of fluid moving parallel to a constant unidirectional flow. The study of fluid flow is crucial because it has a wide range of applications in both engineering and science. Ludwig Prandtl^42^ put forward the concept of the boundary layer. The no-slip condition was assumed at the surface and the flow was inviscid outside the boundary layer. It was shown that the Navier stokes equation can be simplified such that it is applicable only to the boundary layer. It is observed that in contrast to the Navier stokes equation which exhibited elliptic behavior, the boundary layer equation was exhibiting parabolic behavior, which results in simplification in computation. Later Heinrich Blasius^43^ developed a similarity model by introducing a similarity variable to the continuity and momentum equations for steady, incompressible, laminar flow of fluid to obtain a non-linear differential equation of order three, known as the Blasius equation. The fluid’s velocity profile in the boundary layer is described by the equation. Numerous scholars have looked into this well-known fluid dynamics equation to determine its analytical and numerical solutions. We can see from the literature a growing interest in solving this equation for assessing the performance of new computational techniques. In 1908, Blasius obtained the exact solution. In 1938, an accurate numerical solution was obtained by Howarth^44^. Till 1999, no analytical solution was available. Liao^45^ obtained an analytical solution for the Blasius problem using the Homotopy Analysis Method. Various methods have been used to solve this equation, a few of the very recent works are listed below.

Marinca et al.^46^ introduced the Optimal Auxiliary Functions Method and concluded that the obtained first-order approximate solution was accurate. Liu^47^ presented a boundary shape function iterative method to solve the Blasius equation after employing Crocco transformation to the differential equation. Zarnan et al.^48^ developed a numerical method by introducing a system of new approximations based on the inverse Laplace transform using the Chebyshev polynomial function matrix of integration. A leaping Taylor’s series method was used to obtain an accurate solution of the Blasius equation by Anil Lal et al.^49^. Khandelwal et al.^50^ introduced the Adomian Mohand transform method which gave numerical values as well as power series close-form solutions. Mutuk^51^ used a feed-forward neural network to solve the Blasius problem using the method proposed by Lagaris et al.^2^. Recently Bararnia^52^ et al. carried out the first study of PINN in solving the problem by exploring the application of PINN in an unbounded domain. The ‘tanh’ activation function was employed as the activation function in this work. Considering the mentioned advantages of wavelets, in this study, the wavelet activation function is utilized to solve the Blasius problem using PINN.

As systems of coupled second order ordinary differential equations are used to simulate numerous problems in science and engineering^55^, we have taken into consideration two numerical examples of this type. We further extend our approach by using it to solve the well-known Burger’s equation, which arises in the modeling of numerous physical phenomena^56,57^. This equation’s analytical and numerical solution has been the subject of extensive research^58^. The main objectives of this study are to apply PINN to solve the Blasius equation using wavelets as an activation function and to extend the method to solve coupled equations and partial differential equations. Different wavelet functions are taken into account and their performances in solving the problems are compared. Additionally, the effect of neural network architecture on the accuracy of the solutions is examined.

## PINN: method to solve differential equations

**Figure 1:**
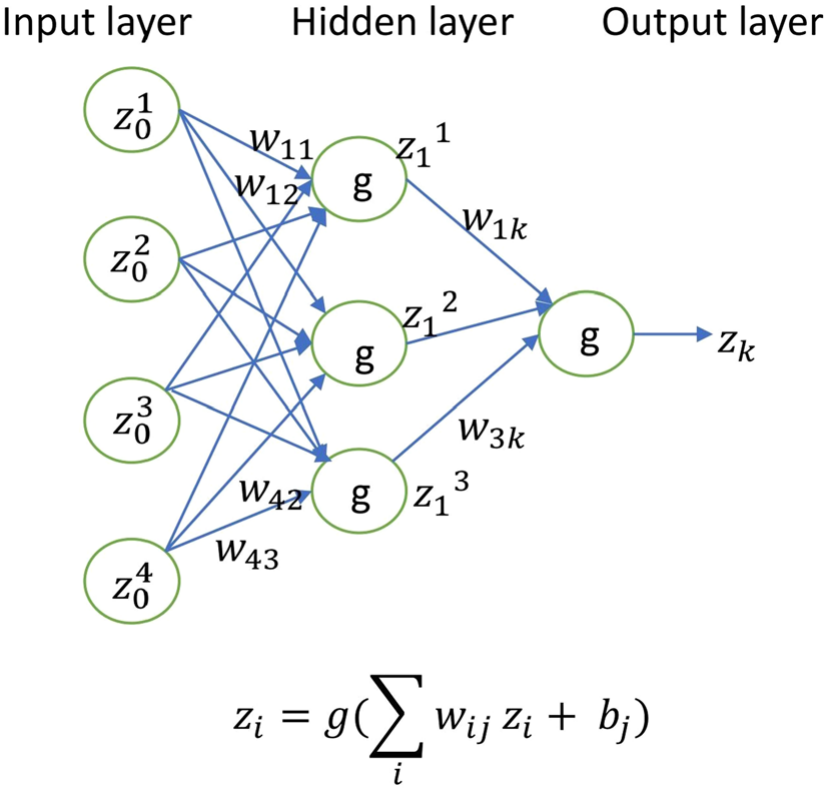
Schematic representation of neural network.

A neural network having interconnected nodes is a mathematical model which works similarly to the neurons in the human brain. An input layer, one or more hidden layers, and an output layer are the three components of a neural network. Data is passed into the input layer, and these data values are passed on to the next layers through each neuron. Finally, a result is produced from the output layer. Schematic diagram of a simple, fully connected neural network is given in Fig. 1. PINNs are related to certain basic properties of neural networks. Hornik^53^ established neural networks as universal function approximators. Hence, we can approximate any function using an appropriate neural network. In this study, feed-forward artificial neural networks are considered. Mathematically, we can write the connection of any two adjacent layers as:

2.1 $$\begin{aligned} z_i = g(W^Tz_{i-1} + b_i) \end{aligned}$$

where $z_0 \in {\mathbb {R}}^{n_0}$ is the input layer, $z_L \in {\mathbb {R}}^{n_L}$ is the output layer and $z_i$ are the hidden layers for $i=1,2, \ldots ,L$, $W_i \in {\mathbb {R}}^{n_{i-1}\times n_i}, b_i \in {\mathbb {R}}^{n_i}$. Here $W_i$ is the weight matrix, $b_i$ is the bias vector and *g* is an activation function. The index of each layer is notated by the subscript *i*. We can consider a neural network as a mapping from an input to an output layer. Now, the neural network is trained by back propagation, where the parameters are adjusted by minimizing the loss function. The loss function (*L*) is defined as the residual of the differential equation, boundary, and initial conditions at specific collocation points within the specified domain of definition. That is, $L= L_f + L_b$, where $L_f$ is the mean squared error of the residual and $L_b$ is the mean squared error of the initial or boundary conditions. “PINN implementation” section defines the particular equations of $L_f$ and $L_b$ for all problems taken into consideration. The loss function is minimized using the gradient descent method. In this process, the gradient of the loss function with respect to the model parameters, i.e., the weights and biases, has to be computed. Furthermore, to determine the derivatives present in the loss function, it is required to compute the derivatives of the network outputs (approximated solution) with respect to the network inputs. These computations are effectively carried out using a technique known as automatic differentiation^54^. Using readily available python libraries, automatic differentiation may be quickly done for any differential equations together with the constraints. Automatic differentiation streamlines the difficult process that would otherwise be required for this most important PINN step. PINN aims to determine the best parameters such that the defined loss function will be minimized. There are also different choices for the activation function and the optimization algorithm. “PINN implementation” section provides these particulars that were employed in this study. Mishra and Molinaro^23^ presented a thorough overview of PINN.

## Problems studied

### Non-linear ordinary differential equation (Blasius equation)

For the steady, incompressible, two-dimensional flow with constant properties, the boundary layer equations is given by^43^:

3.1 $$\begin{aligned}{} & {} \frac{\partial u}{\partial x} + \frac{\partial v}{\partial y} = 0 \end{aligned}$$

3.2 $$\begin{aligned}{} & {} u\frac{\partial u}{\partial x} + v\frac{\partial u}{\partial y} = -\frac{1}{\rho }\frac{\partial p}{\partial x} + \nu \frac{\partial ^2 u}{\partial ^2 y} \end{aligned}$$

3.3 $$\begin{aligned}{} & {} u\frac{\partial v}{\partial x} + v\frac{\partial v}{\partial y} = -\frac{1}{\rho }\frac{\partial p}{\partial y} + \nu \frac{\partial ^2 v}{\partial ^2 y} \end{aligned}$$

Considering the flow along x-axis, Blasius introduced a similarity variable $\eta$ in such a way that,

$$\begin{aligned} \eta = \frac{y}{\delta (x)} = y \sqrt{\frac{U}{\nu x}}, \quad \psi = \sqrt{\nu Ux}f(\eta ) \end{aligned}$$

where $\delta (x) \propto \sqrt{\nu x/U}$ is the thickness of the boundary layer, $\psi$ is the stream function, *U* is the free stream velocity. $f(\eta )$ is the normalized function of $\eta$, where $f(\eta ) \propto \psi$. Velocity components can be obtained from these and its substitution in the x- momentum equation give rise to the Blasius equation^43^ given by:

3.4 $$\begin{aligned} 2f''' + ff'' = 0 \end{aligned}$$

The boundary conditions, $u(x,0) = 0 = v(x,0), u(x,\infty ) = U$ becomes,

3.5 $$\begin{aligned} f'(0) = 0, f(0) = 0, f'(\infty ) = 1 \end{aligned}$$

We demonstrate the versatility of the suggested method by extending its application to solve both linear coupled equations, non-linear coupled equations and a partial differential equation in addition to the Blasius equation. Additionally, we compare the numerical results for these problems with those available in the literature.

### Coupled differential equations

#### Linear coupled equation

Consider the linear coupled equation^55^ given by:

3.6 $$\begin{aligned}{} & {} u'' + xu + 2v' = u_1 \nonumber \\{} & {} u + v'' + 2v = u_2 \end{aligned}$$

with the boundary conditions:

3.7 $$\begin{aligned}{} & {} u'(0) + u(0) = 1 \nonumber \\{} & {} v'(1) + v(1) = cos(1) + sin(1) \nonumber \\{} & {} u(1) = 2, v'(0) =1 \end{aligned}$$

where *u* and *v* are function of *x*, $0 \le x \le 1$, $u_1 = x^3 + x^2 + 2 + 2 cos(x)$ and $u_2 = x^2 + x + sin(x)$

#### Non-linear coupled equation

Consider the non-linear coupled equation^55,59^ given by:

3.8 $$\begin{aligned}{} & {} u'' + xu + 2xv + xu^2 = u_3 \nonumber \\{} & {} x^2u + v' + v +sin(x)v^2 = u_4 \end{aligned}$$

with the boundary conditions:

3.9 $$\begin{aligned}{} & {} u(0)=u(1)=0 \nonumber \\{} & {} v(0)=v(1)=0 \end{aligned}$$

where *u* and *v* are function of *x*, $0 \le x \le 1$, $u_3 = 2xsin(\pi x) + x^5 - 2x^4 + x^2 - 2$ and $u_4 = x^3(1-x) + sin(\pi x)(1 + sin(x) sin(\pi x))+ \pi cos(\pi x)$^55^.

### Partial differential equations

We consider the Burger’s equation^56^ in one dimension given in the Eq. (3.10) for two different cases:

3.10 $$\begin{aligned} u_t + uu_x = \nu u_{xx} \end{aligned}$$

#### Case 1

Burgers equation^66^ with the initial condition:

3.11 $$\begin{aligned} u(0,x) = sin(\pi x) \end{aligned}$$

and boundary conditions:

3.12 $$\begin{aligned} u(t,0)=u(t,1)=0 \end{aligned}$$

where $t>0$, $0<x<1$, $\nu > 0$ is the coefficient of the kinematic viscosity, and in this example we consider $\nu = 0.1$

#### Case 2

Burgers equation^3^ with the initial condition:

3.13 $$\begin{aligned} u(0,x) = - sin(\pi x) \end{aligned}$$

and boundary conditions:

3.14 $$\begin{aligned} u(t,-1)=u(t,1)=0 \end{aligned}$$

where $t>0$, $-1<x<1$, $\nu > 0$ is the coefficient of the kinematic viscosity, and in this example we consider $\nu = \frac{10^{-2}}{\pi }$

### PINN implementation

A neural network’s optimum design is important for obtaining an accurate solution. In this study, we look into the role of significant factors such as the number of collocation points, hidden layers, and neurons for effective learning of the model. A successful training procedure also depends on the optimization method and activation function that is chosen. The technique used to initialize the unknown parameters and the optimizer’s learning rate are also important factors in the training process. We now outline the implementation details that are used.

For the Blasius equation, the loss function is given by $L = L_f + L_b$, where

3.15 $$\begin{aligned} L_f= & {} \sum _{i=1}^{N_f}\left\{ (2f'''(x_i) + f(x_i)f''(x_i))^2\right\} /N_f \end{aligned}$$

3.16 $$\begin{aligned} L_b= & {} \sum _{i=1}^{N_b}\left\{ (f(x_{0i})^2 + (f'(x_{0i})))^2 + (f'(x_{1i}) - 1)^2\right\} /N_b \end{aligned}$$

For the linear coupled equation, the loss function is given by $L = L_f + L_b$, where

3.17 $$\begin{aligned} L_f= & {} \sum _{i=1}^{N_f}[(u''(x_i) + x_iu(x_i) + 2v'(x_i) - u_1(x_i))^2 + (u(x_i) + v''(x_i) + 2v(x_i) - u_2(x_i))^2]/N_f \end{aligned}$$

3.18 $$\begin{aligned} L_b= & {} \sum _{i=1}^{N_b}[(u'(x_{0i}) + u(x_{0i}) - 1)^2 + (v'(x_{1i}) + v(x_{1i}) - cos(1) - sin(1))^2 \nonumber \\{} & {} \quad + (u(x_{1i}) - 2)^2 + (v'(x_{0i}) - 1)^2]/N_b \end{aligned}$$

For the non-linear coupled equation, the loss function is given by $L = L_f + L_b$, where

3.19 $$\begin{aligned} L_f= & {} \sum _{i=1}^{N_f}[(u''(x_i) + x_iu(x_i) + 2x_iv(x_i) + x_iu(x_i)^2 - u_3(x_i))^2 \nonumber \\{} & {} \quad + (x_i^2u(x_i) + v'(x_i) + v(x_i) + sin(x_i)v(x_i)^2 - u_4(x_i))^2]/N_f \end{aligned}$$

3.20 $$\begin{aligned} L_b= & {} \sum _{i=1}^{N_b}[(u(x_{0i}))^2 + (v(x_{0i}))^2 + (u(x_{1i}))^2 + (v(x_{1i}))^2]/N_b \end{aligned}$$

For the partial differential equation (case 1), the loss function is given by $L = L_f + L_b$, where

3.21 $$\begin{aligned}{} & {} L_f = \sum _{i=1}^{N_f}\left\{ (u_t(t_i,x_i) + u(t_i,x_i) u_x(t_i,x_i) - \nu u_{xx}(t_i,x_i))^2\right\} /N_f \end{aligned}$$

3.22 $$\begin{aligned}{} & {} L_b = \sum _{i=1}^{N_b}\left\{ u(t_i,x_{0i})^2 + (u(t_i,x_{1i}))^2 + (u(t_{0i},x_i) - sin(\pi x_i))^2\right\} /N_b \end{aligned}$$

For the partial differential equation (case 2), the loss function is given by $L = L_f + L_b$, where

3.23 $$\begin{aligned}{} & {} L_f = \sum _{i=1}^{N_f}\left\{ (u_t(t_i,x_i) + u(t_i,x_i) u_x(t_i,x_i) - \nu u_{xx}(t_i,x_i))^2\right\} /N_f \end{aligned}$$

3.24 $$\begin{aligned}{} & {} L_b = \sum _{i=1}^{N_b}\left\{ u(t_i,x_{0i})^2 + (u(t_i,x_{1i}))^2 + (u(t_{0i},x_i) + sin(\pi x_i))^2\right\} /N_b \end{aligned}$$

The solution of the differential equation is approximated by the neural networks and hence the solution *f*, *u*, *v* are now the function of *x*, weights and biases. $L_f$ denotes penalization of the residuals of the differential equation and $L_b$ denotes that of the boundary conditions. $N_b$ and $N_f$ denotes the number of collocation points used. The *ith*- component of the input vector for the residual term is represented by $x_i$, $t_i$, while the *ith*components for the boundary terms are represented by $x_{0i}$, $x_{1i}$, $t_{0i}$. The values of these quantities are assigned depending on the domain of the definition of the problem.

Each problem has a different optimal number of collocation points that must be taken into account. Without any preprocessing, the collocation points for the residual term, $x_i$ and $t_i$ are generated from a uniform distribution. By selecting various values for $N_f$ and $N_b$, we attempt to ascertain the impact of the choice of $N_f$ and $N_b$ on the error of the model. Ideally, the accuracy of the prediction would improve as the number of collocation points is increased because more the points, less the error would be. This particular trend observed in research works is concluded to be an important advantage of PINN^3^.

Numerous research studies have shown that the number of hidden layers and neurons also affects approximation errors. To find the optimal number, we varied these and looked at the approximation errors. Because these quantities are problem-specific, trial and error were used to determine the best value.

**Figure 2:**
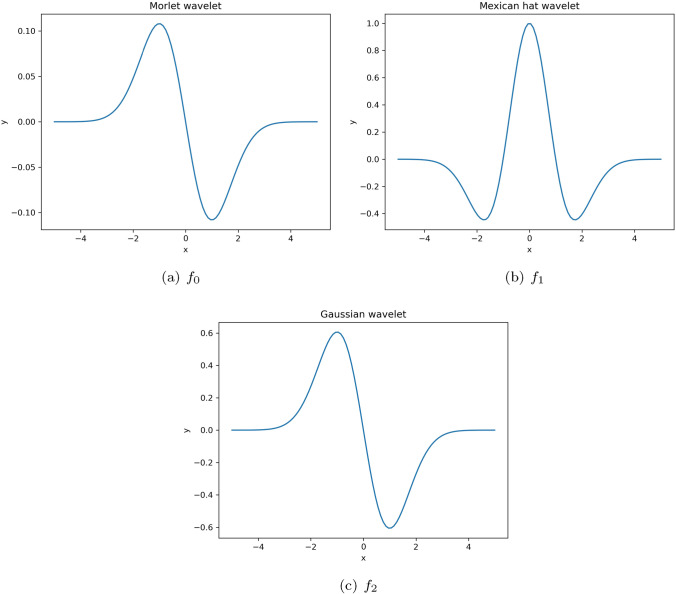
Diagrams of the used wavelet functions.

Another crucial aspect of training process is the role of activation function. We examine the performance of three distinct wavelet activation functions. The equation and the illustration (Fig. 2) of wavelet activation function used in this study including Morlet function ($f_0$), Mexican hat function ($f_1$), and Gaussian wavelet function ($f_2$) are given below.

3.25 $$\begin{aligned}{} & {} f_0(x) = cos(\frac{7}{4}x) e^{-\frac{x^2}{2}} \end{aligned}$$

3.26 $$\begin{aligned}{} & {} f_1(x) = (1-x^2)e^{\frac{-x^2}{2}}\end{aligned}$$

3.27 $$\begin{aligned}{} & {} f_2(x) = -xe^{\frac{-x^2}{2}} \end{aligned}$$

Although there are many options for optimizers, the Adam algorithm was utilized, which is observed to have a lot of advantages^60^. The Adam optimization is a combination of two stochastic gradient descent techniques, namely adaptive gradient and root mean squared propagation, and hence has the advantages of both the algorithms.

The choice of learning rate affects the convergence of techniques that utilize gradient descent approaches. In this study, we utilize the decaying learning rate i.e. the network is first trained with a high learning rate, which is then gradually reduced. This is believed to be advantageous since a higher learning rate at the beginning will improve the optimization algorithm’s search for optimal value, and the decaying rate will aid in the algorithm’s convergence towards the minima. Experimentally, this process is seen to support optimization^8,61^. Additionally, we employ full batch gradient descent, which means that we only modify the weights once the entire training set has been sent to the network.

According to the studies, effective initialization of parameters could result in fewer iterations needed for the training process and for the optimizing algorithm to avoid being stuck at local minima. So, Xaviers’ technique^62^ is employed rather than initializing the parameters arbitrarily. If the values of the outputs from each layer kept going up or down, the training process would be delayed because the gradients would become excessively small or large during the back propagation. The output from each layer’s activation function should have the same variance in order to avoid this. This is the main idea of Xavier initialization.

**Figure 3:**
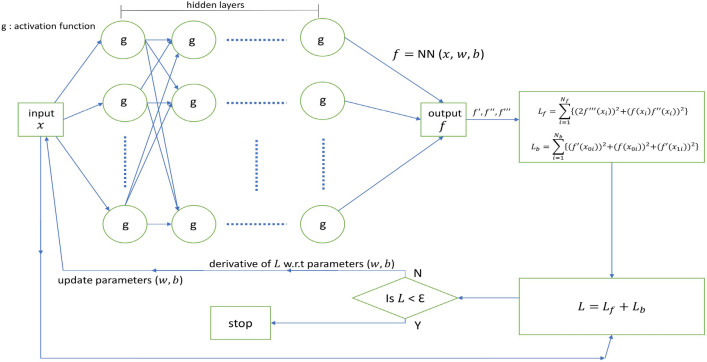
Schematic representation of PINN for Blasius viscous flow problem.

**Figure 4:**
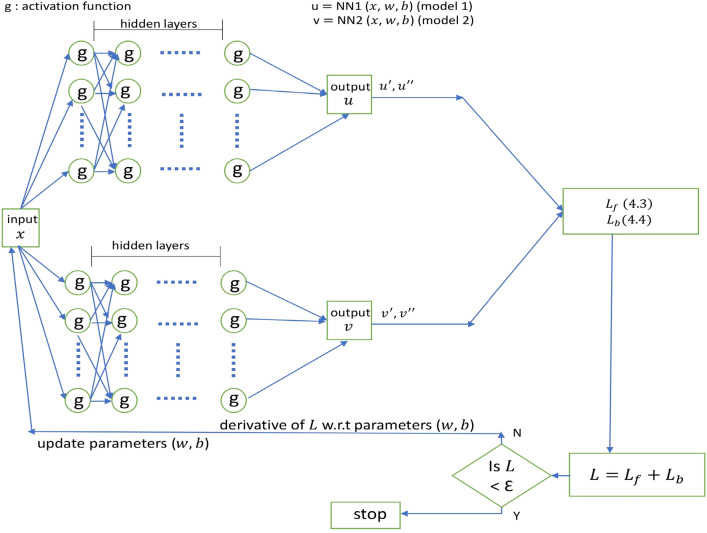
Schematic representation of PINN for coupled equation.

Tensorflow is employed for all implementations. Computations were performed on a GPU server consisting of 2 graphics cards (of 24 GB memory each) that belong to the ZOTAC GeForce RTX 3090, which has a total memory of 128 GB along with 4TB SSD storage. The model’s training took approximately 66 s, 58 s, 77 s, and 37 s for Blasius, coupled linear, coupled non-linear, and Burger’s equations, respectively. Figure 3 provides the schematic representation of the PINN framework for the Blasius viscous flow problem. Due to the fact that different functions are approximated by different neural network designs, for the coupled equation we also tried employing two independent models for two functions in parallel rather than one model to approximate both functions. Figure 4 provides the schematic representation of the PINN framework for the considered coupled equations. Two evaluation indices, the absolute error and the relative $L^2$ error^20^, are used in order to compare the results obtained from the proposed method with those previously published. They are defined as follows:

3.28 $$\begin{aligned}{} & {} \text {Absolute error} = |\widehat{f_k} - f_k|\end{aligned}$$

3.29 $$\begin{aligned}{} & {} \text {Relative} \, L^2 \text {error} = \sqrt{\frac{ \sum _{k=1}^N (\widehat{f_k} - f_k)^2}{\sum _{k=1}^N (f_k)^2}} \end{aligned}$$

where $\widehat{f_k}$ denotes the *kth* approximated solution, $f_k$ denotes the *kth* exact solution and *N* specifies the number of data points considered.

## Results and discussion

**Figure 5:**
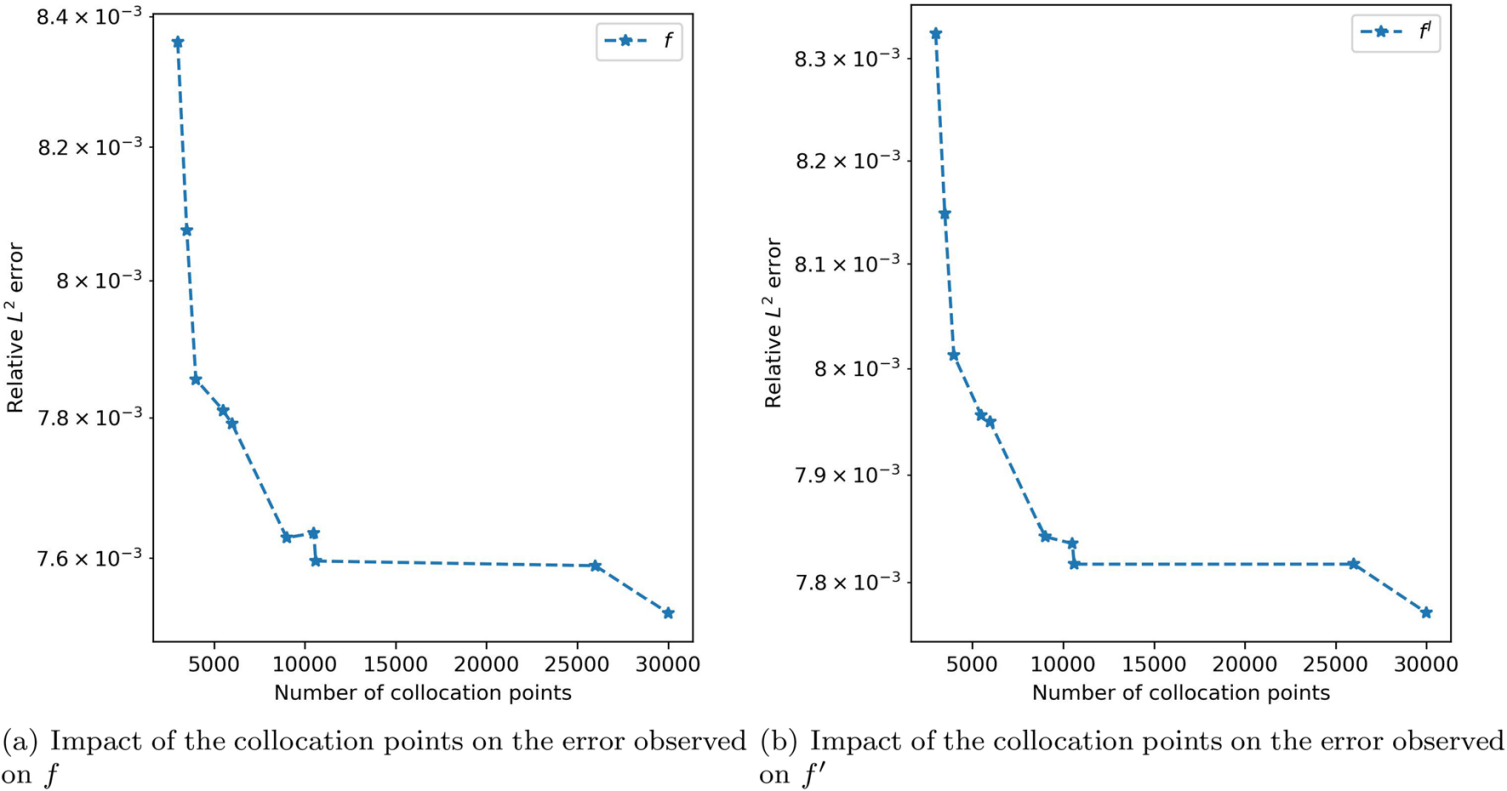
Collocation points versus error.

The above-mentioned implementation was employed to determine the solution for the Blasius equation, linear coupled equation, non-linear coupled equation and partial differential equations. We now discuss the impact of few hyper parameters of neural network in approximating the solution of Blasius equation. Different numbers of collocation points were used and the model accuracy was tested. It was observed that as the collocation points increased, errors were decreased. However, for this particular problem, after a certain number of collocation points, in this case, 10,000, by adding more collocation points, there was a decrease in the training speed and also there was only a small decrement in error. Hence, we choose $N_f = 10,000$. Figure 5 depicts the trend of the relative $L^2$ error for *f* and $f'$ as the number of collocation points increases. Increasing the number of collocation points does not appear to have significant effects on the error. $N_u$ denotes the number of initial and boundary data used for training, and it is chosen to be 50 on a trial-and-error basis. However, altering the value of $N_u$ doesn’t appear to have a substantial effect on the error.

For computational easiness, the right boundary is chosen to be 10 and the collocation points are generated from the uniform distribution within the domain defined for the problem. Because the domain of the problem under consideration is unbounded, the infinity boundary must be numerically approximated by a large number. Any value less than 5 would not be ideal for the considered problem, as the derivative of the solution starts showing asymptotic behavior only after 5. Hence, any value less than 5 cannot be considered the right boundary. And any values greater than 10 also need not be considered because the derivative of the solution behaves asymptotically towards the known boundary condition which is $f'(\infty ) = 1$. When it comes to training a neural network, we have chosen the training data over a domain. For that, we have tried approximating the solution on different right boundaries, for values smaller and larger than 10. It was observed that the loss function was minimized only when the right boundary was chosen to be 10 for the hyper parameters considered in this study. It was achieved by dividing the domain into two sub domains, [0,5] and [5,10], which only interact with one another through their common boundary. In each sub domain, the same neural network architecture was employed, and combining the solutions to each sub problem related to the full domain yields the final solution.

**Figure 6:**
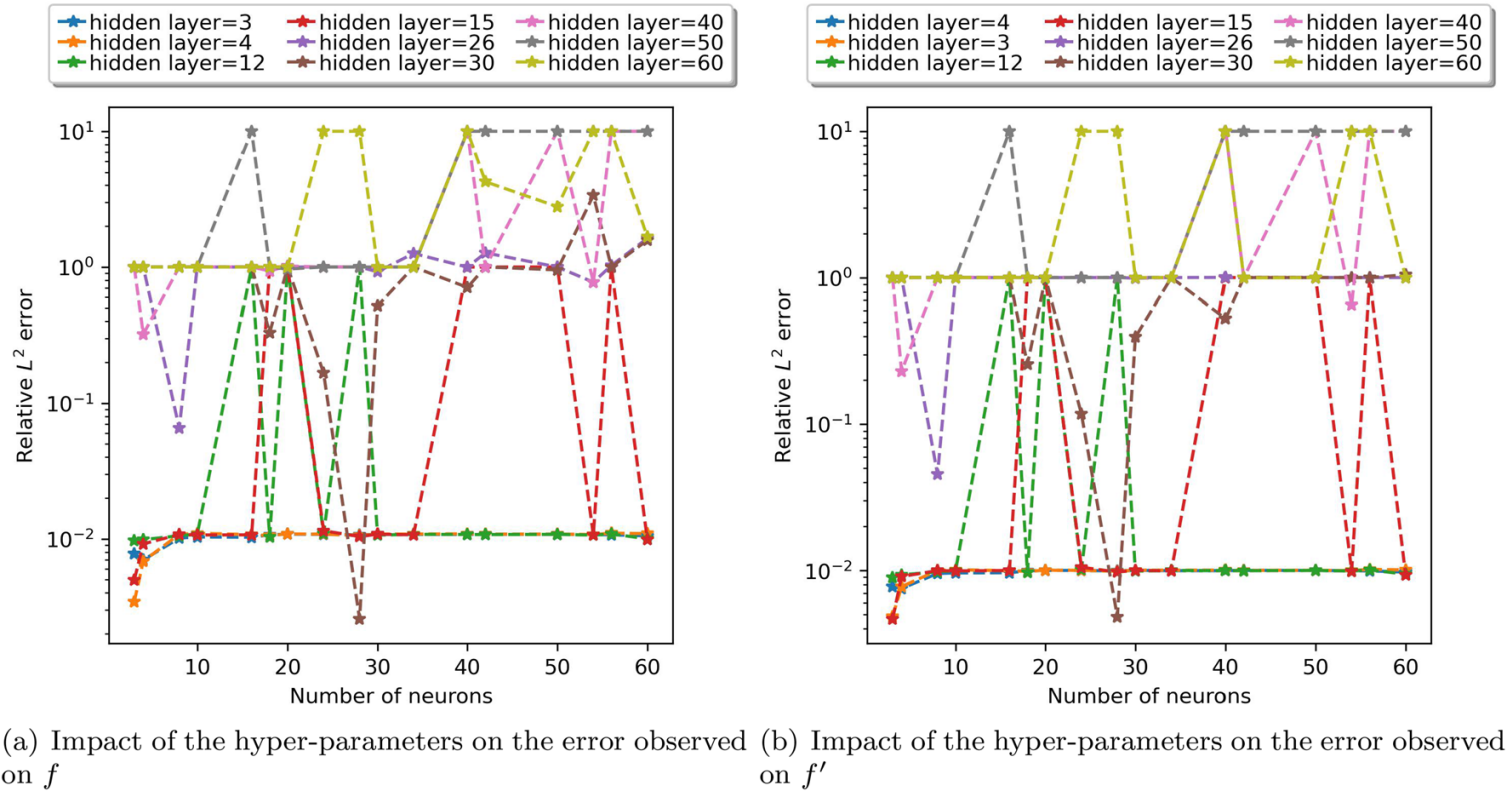
Number of neurons versus error.

**Figure 7:**
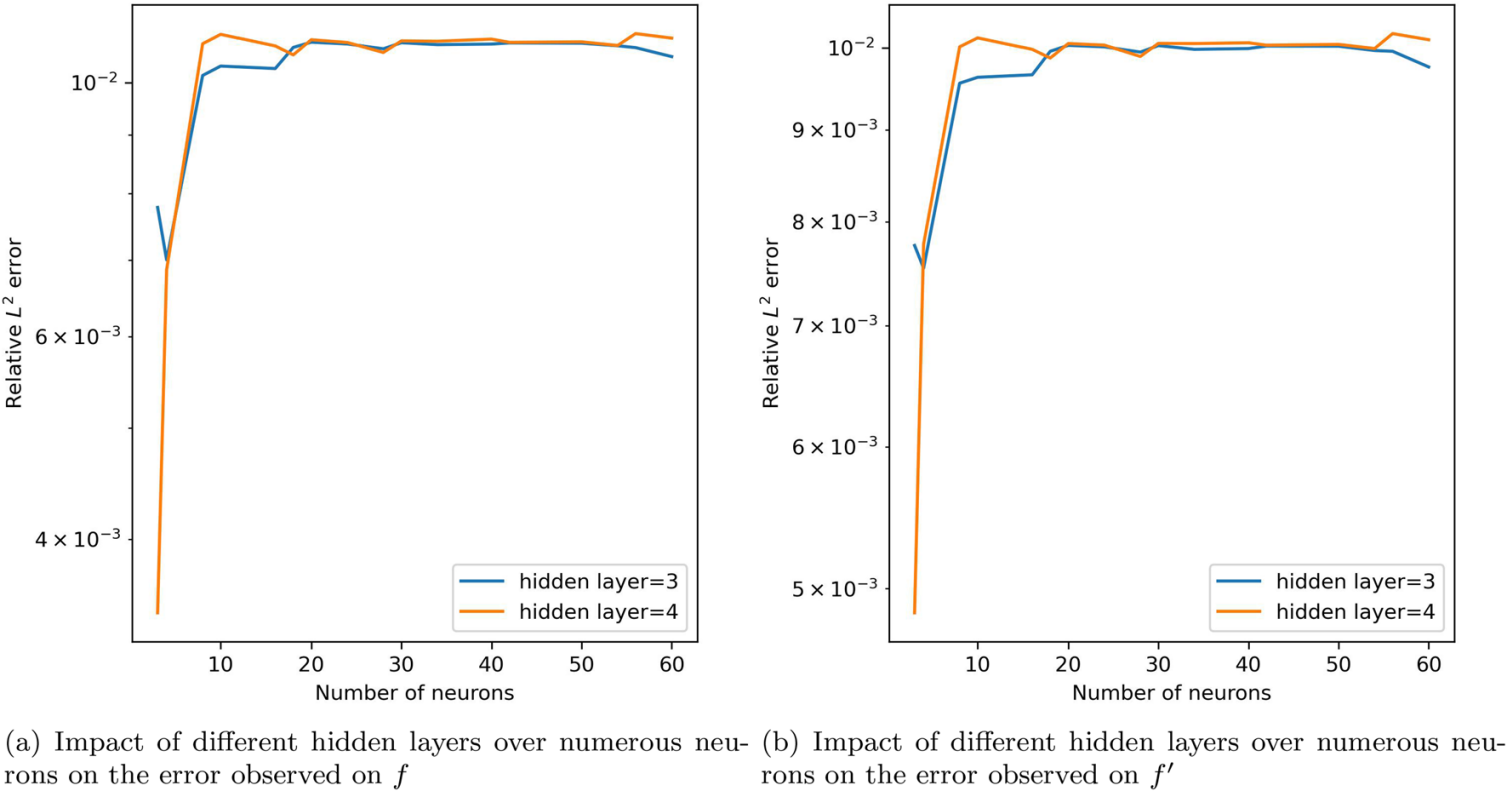
Number of neurons versus error (when number of hidden layers equals 3 and 4).

By adjusting the number of hidden layers and neurons from 3 to 60, relative $L^2$ errors were observed. Figure 6 depicts the effect of increasing the number of neurons on both *f* and $f'$ over some fixed hidden layers. The errors for hidden layers 3 and 4 are similar and give the least error with a smaller number of neurons. The trend of errors for both *f* and $f'$ on these hidden layers by varying the number of neurons is shown in Fig. 7 for further understanding. Among the values of the hyperparameters considered, these errors are minimum and there is only a slight variation while changing the neurons. We can observe the error reaching minimum for certain neurons for hidden layer 12, 15 and 30 as well. However, to keep the model simple and since there isn’t any significance difference in the minimum error achieved with relatively larger network, 4 hidden layers and 4 neurons in each hidden layer were selected. It was also observed that as the number of hidden layers was increased, more than 30, the model was not learning properly as the value of loss function was not converging. For showing this trend in plots, the large value obtained was replaced with 10. And it can be observed for hidden layers 40, 50 and 60, the errors are higher in comparison with the lesser number of hidden layers.

Three wavelet functions were employed as activation functions, and the effectiveness of each was evaluated in comparison to the ‘tanh’ activation function. Additionally, the proposed method was compared with the wavelet Galerkin method (WGM)^63^ and is considered as the benchmark solution for the comparison of the results. The results of which were observed to be consistent with Howarth’s results. The proposed method was also compared with the Differential Transformation Method (DTM)^64^. The benefits of wavelets, such as their multi-resolution and localization capabilities, have contributed to the widespread use of WGM. However, it comes with a few drawbacks^65^, like its inability to handle many general boundary conditions, making the method complicated. It should be noted that physics-informed neural networks using wavelet as an activation function are simpler and more straightforward for handling this particular problem than the WGM, allowing us to apply the suggested method to complex problems where the WGM is challenging to use.

**Figure 8:**
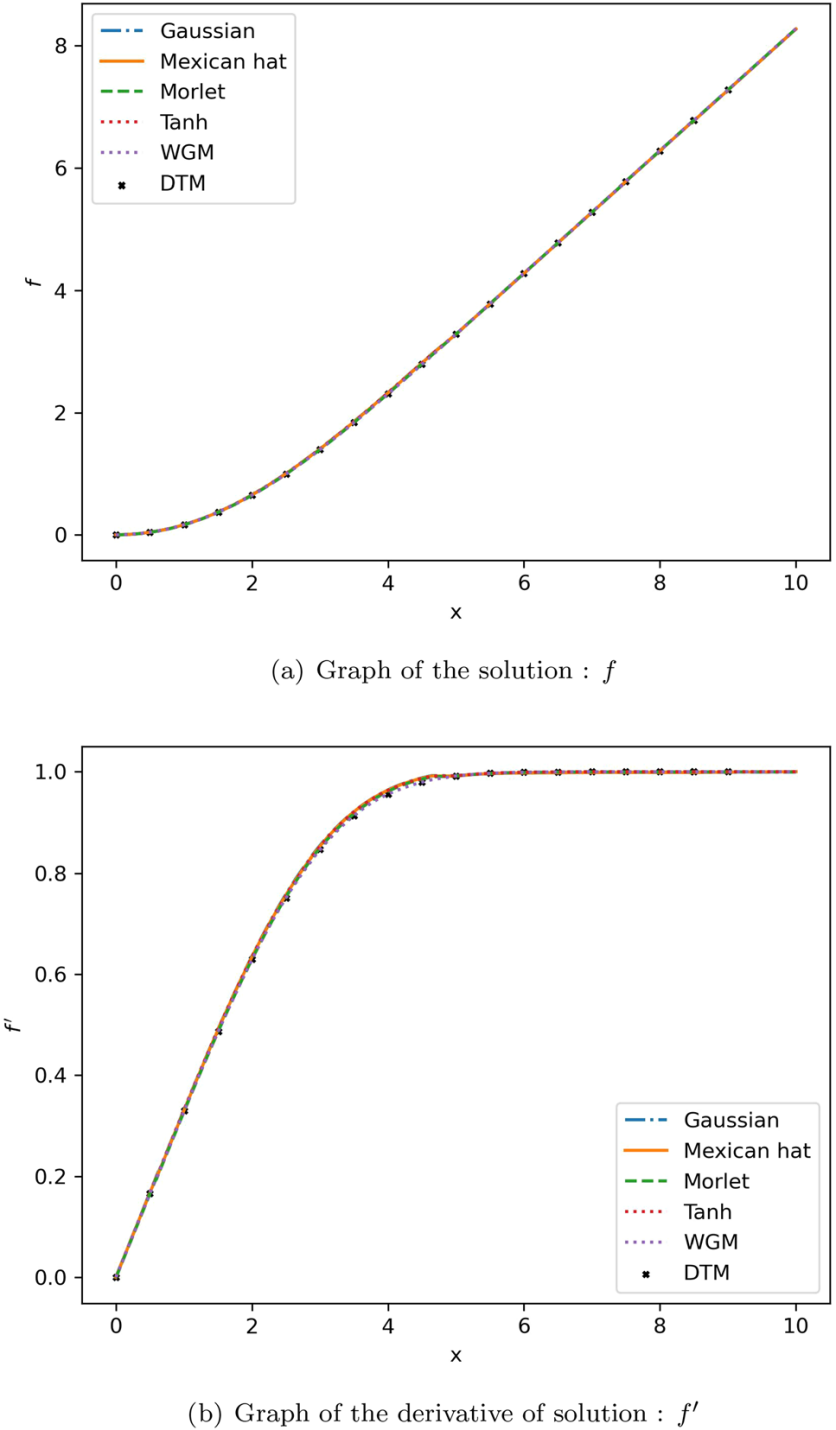
Comparison of the results of proposed method with different activation functions and existing results for the Blasius equation.

Now we will discuss the impact of using different activations. Figure 8 graphically illustrates the results of the comparison between the suggested approach and the existing methods.

The absolute error of the obtained solution using the proposed method with that of the WGM is provided in Table A1 in online Appendix.

**Figure 9:**
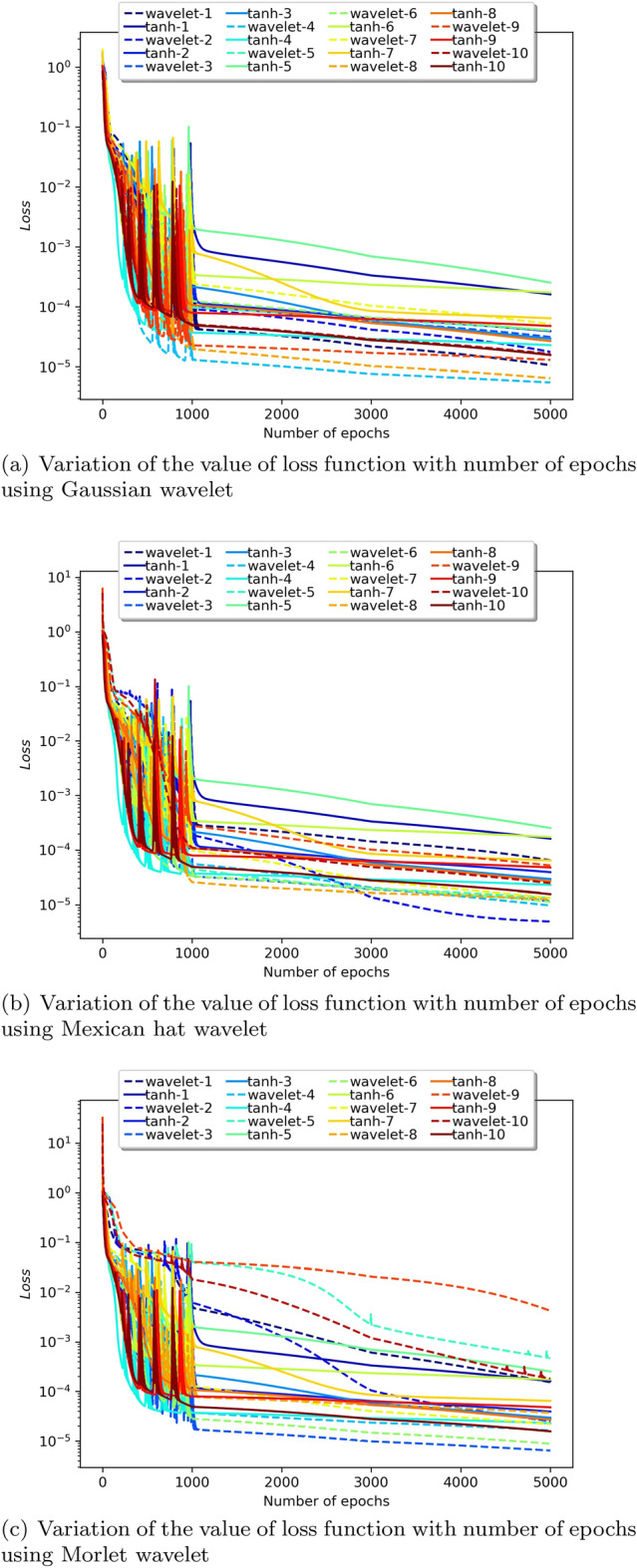
Epoch versus Loss of the proposed method against ‘tanh’ over 10 instances for the Blasius equation.

To determine the unknown parameters, the network is trained by minimizing a loss function. For each epoch, the loss function is calculated for this purpose. The optimizer alters the weights and biases during training in order to lower the overall value of the loss function at each epoch. And so, ideally, the value of the loss function is expected to decrease with increasing epochs. Figure 9 plots the number of epochs against the loss value for all the considered wavelet activation functions with ‘tanh’ run over 10 instances. The ten different instances were considered by varying the initialization of model parameters and the input. In contrast to the ‘tanh’ activation function, PINN utilizing certain wavelet produces lower loss values. However, almost all activation functions appear to converge around 1000 epochs and past that decrease at a similar rate. Table 1 provides the average and standard deviation (SD) of relative $L^2$ error of the PINN utilizing various activation functions against WGM for ten different instances.

We can see that when estimating the solution to the Blasius equation, the Mexican hat wavelet as an activation function provided the least relative $L^2$ error. Each wavelet function can be seen to produce a distinct error; as a result, we may infer that selecting the best wavelet for a given problem is an important factor to consider.

As previously indicated, this method uses a piecewise decline of the learning rate; the learning rate chosen is 0.01 for the first 1000 epochs, 0.001 for epochs starting at 1000, and 0.0005 for epochs exceeding 3000. We can observe that after 1000 epochs, the loss value consistently decreases (Fig. 9), suggesting the benefit of tweaking the learning rate as opposed to having a fixed learning rate.

**Table 1:** Average and SD of relative $L^2$ error of the proposed method in solving Blasius equation using different activation function compared against WGM over 10 instances.

Activation	Mean		SD	
	*f*	$f'$	*f*	$f'$
Mexican hat	6.85E−03	6.59E−03	5.63E−04	3.64E−04
Gaussian	6.97E−03	6.63E−03	6.36E−04	3.67E−04
tanh	6.91E−03	6.65E−03	1.05E−03	6.72E−04
Morlet	1.58E−02	1.30E−02	3.26E−02	2.30E−02

The proposed method is extended to solve linear and non-linear coupled equations. Next, the impact of neurons and hidden layers on solving coupled equations is discussed. It was observed that adding more neurons while keeping the hidden layers constant improved accuracy in some cases, whereas increasing more and more neurons was observed to reduce accuracy. Furthermore, adding more hidden layers did not enhance the results. A general trend could not be seen for the case of the considered coupled equations, so by trial and error, we were able to determine the number of neurons and hidden layers that would give the desired accuracy.

Since different functions are approximated by different neural network architectures, we also attempted to utilize two separate models for two functions, *u* and *v*, in parallel rather than using one model to approximate both functions, *u* and *v*. It was found that a simpler architecture and the requisite accuracy were achieved when two distinct models were used to approximate *u* and *v*. In the case of linear coupled differential equation, we have considered two different models for comparing the results with different activation functions. However, the relative $L^2$ error of utilizing a single model in case of linear coupled equation is given in Table  A5 in online Appendix. In the case of non-linear coupled equation, when compared to two different models, a single model for both *u* and *v* provided the required accuracy, which could be attributed to the similar behavior of the solutions to both *u* and *v* in this example.

**Figure 10:**
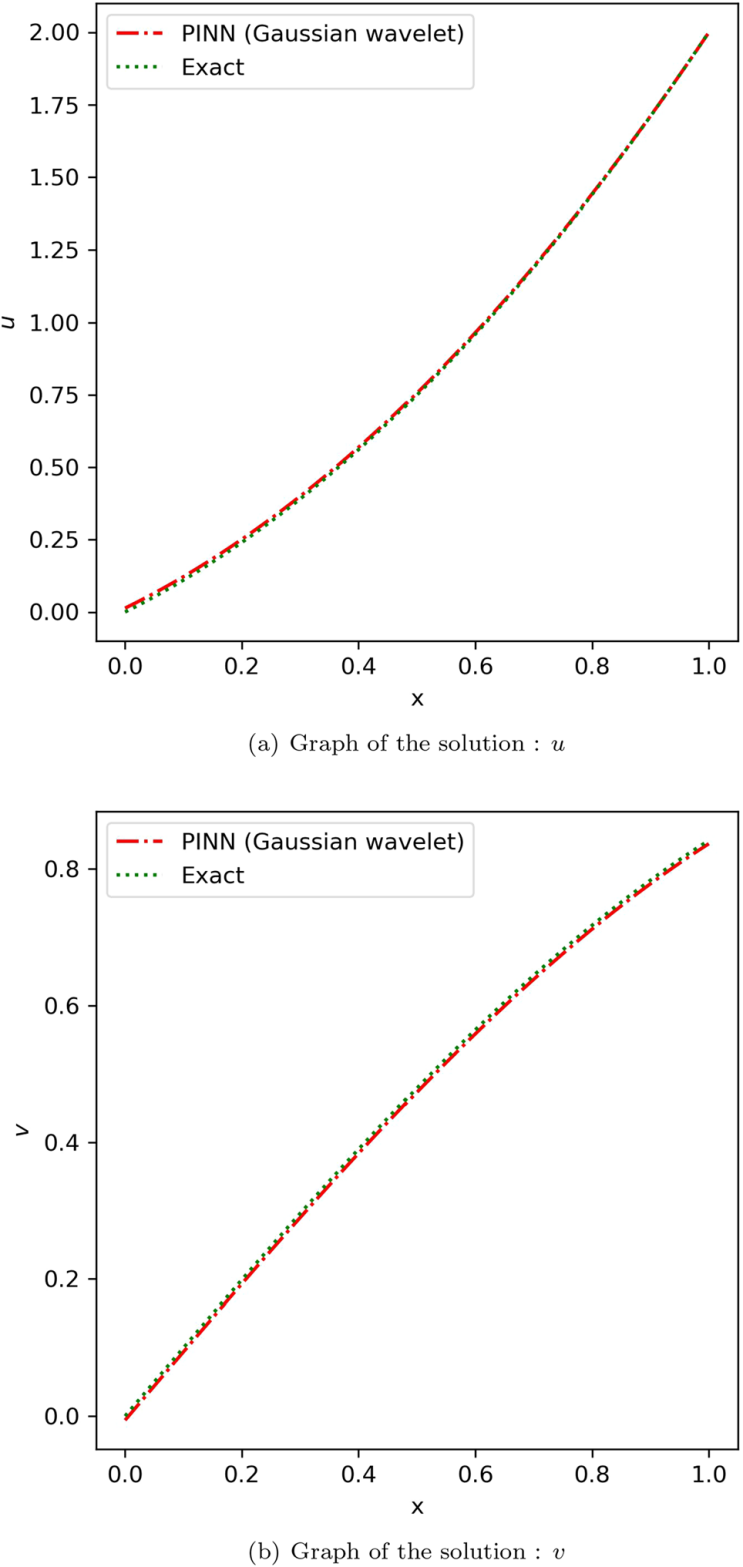
Comparison of the results of proposed method using Gaussian wavelet with the exact solution for the linear coupled equation.

**Figure 11:**
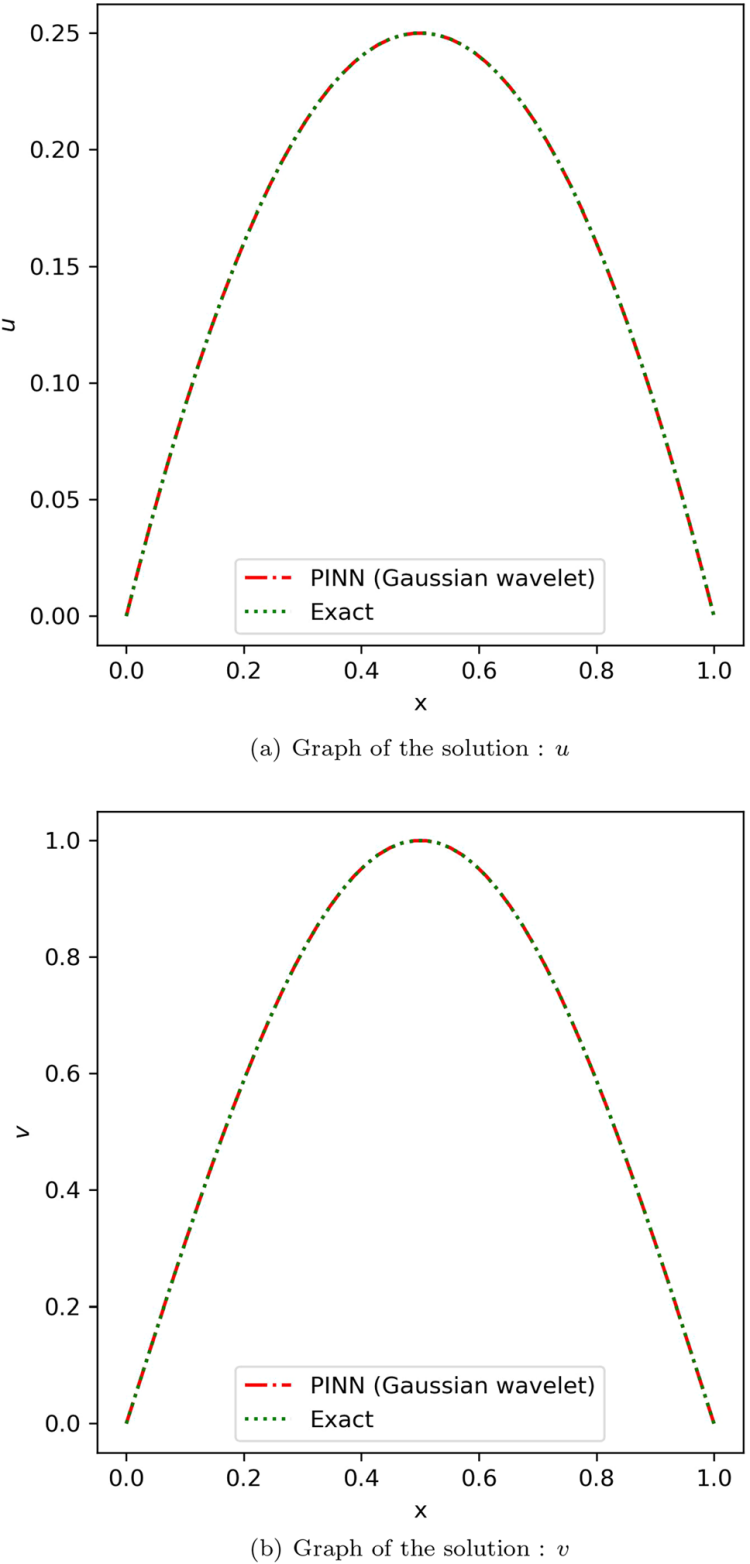
Comparison of the results of proposed method using Gaussian wavelet with the exact solution for the non-linear coupled equation.

When choosing collocation points, coupled equations also showed the same deductions from the Blasius equation. An effective model was obtained from the below mentioned choice of hyperparameters in the case of the linear coupled equation. For the model approximating *u*, the number of neurons was 20 and the number of hidden layers was 4, while for the model approximating *v*, the number of neurons was 10 and the number of hidden layers was 5. In the case of non-linear coupled equations, using a single model with number of neurons = 8 and number of hidden layers = 8 was effective. The absolute error of the obtained solution using the proposed method with that of the analytical solution observed for linear coupled equation is tabulated in Table A2 in online Appendix and that of non-linear coupled equation is tabulated in Table A3 in online Appendix. The comparison of the proposed method with that of the exact solution^55^ for linear and non-linear coupled equations is illustrated in Figs. 10 and 11 respectively. Table 2 provides the average and standard deviation (SD) of relative $L^2$ error of the PINN utilizing various activation functions against the analytical solution for ten different instances for linear coupled equation and Table 3 provides the same for non-linear coupled equation. In the case of linear coupled equation and non-linear coupled equation, it can be observed that, comparatively, the Mexican hat wavelet outperforms other activation functions. Hence, by choosing an ideal wavelet as an activation function for specific problems, we can obtain the solution using PINN with improved accuracy.

**Table 2:** Average and SD of relative $L^2$ error of the proposed method in solving linear coupled equation using different activation function compared against the analytical solution over 10 instances.

Activation	Mean		SD	
	*u*	*v*	*u*	*v*
Mexican hat	1.33E−02	1.79E−02	1.01E−02	1.32E−02
Gaussian	1.38E−02	2.02E−02	1.25E−02	1.79E−02
tanh	4.63E−02	6.72E−02	2.13E−02	3.08E−02
Morlet	3.42E−02	5.04E−02	1.92E−02	2.79E−02

**Table 3:** Average and SD of relative $L^2$ error of the proposed method in solving non-linear coupled equation using different activation function compared against the analytical solution over 10 instances.

Activation	Mean		SD	
	*u*	*v*	*u*	*v*
Mexican	2.46E−04	9.49E−05	2.09E−04	3.84E−05
Gaussian	4.27E−04	2.06E−04	5.34E−04	7.41E−05
tanh	3.78E−04	2.18E−04	4.98E−04	5.67E−05
Morlet	5.99E−03	3.96E−04	1.06E−02	7.11E−04

**Figure 12:**
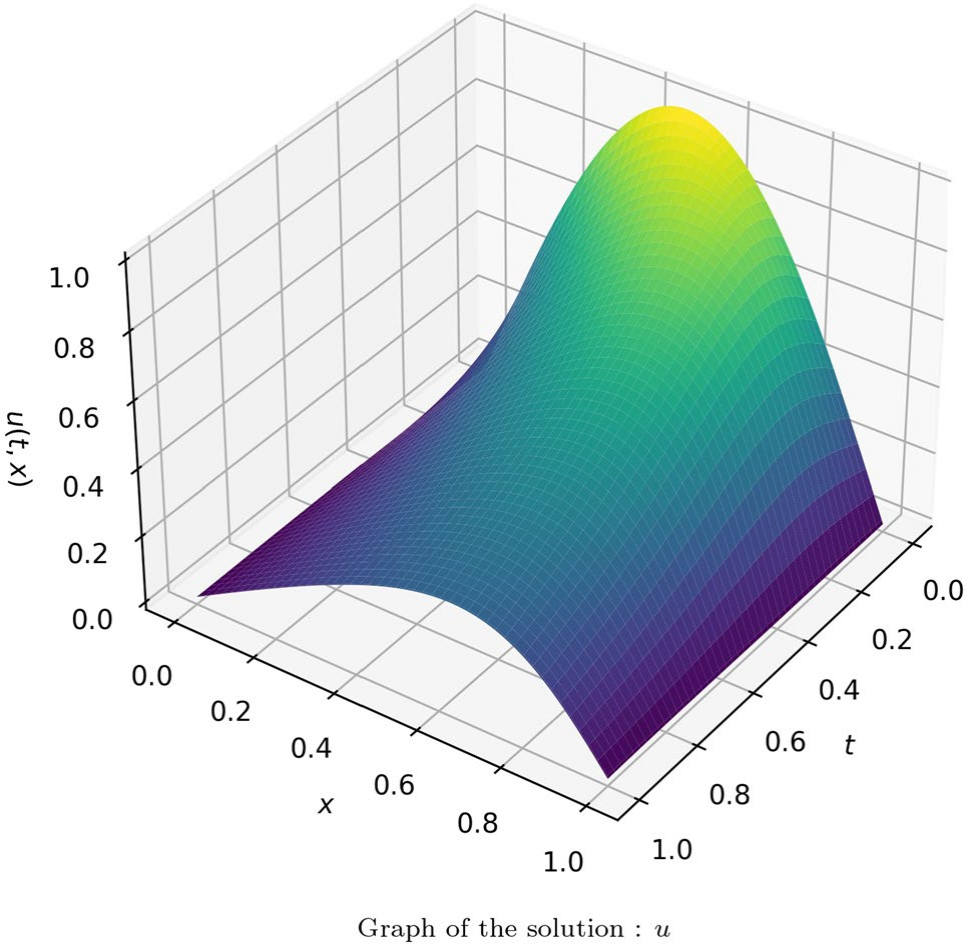
Solution obtained for the Burger’s equation (case 1) using the proposed method with Gaussian wavelet.

**Figure 13:**
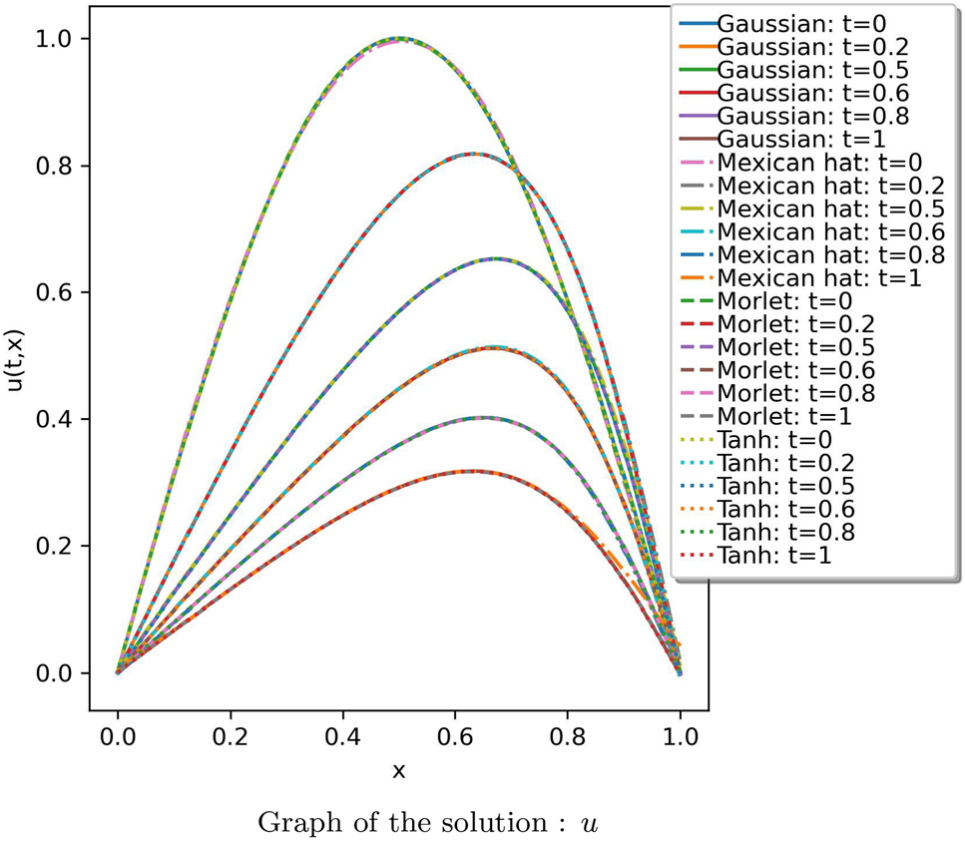
Comparison of the results of proposed method against the ‘tanh’ activation function for different values of t.

**Figure 14:**
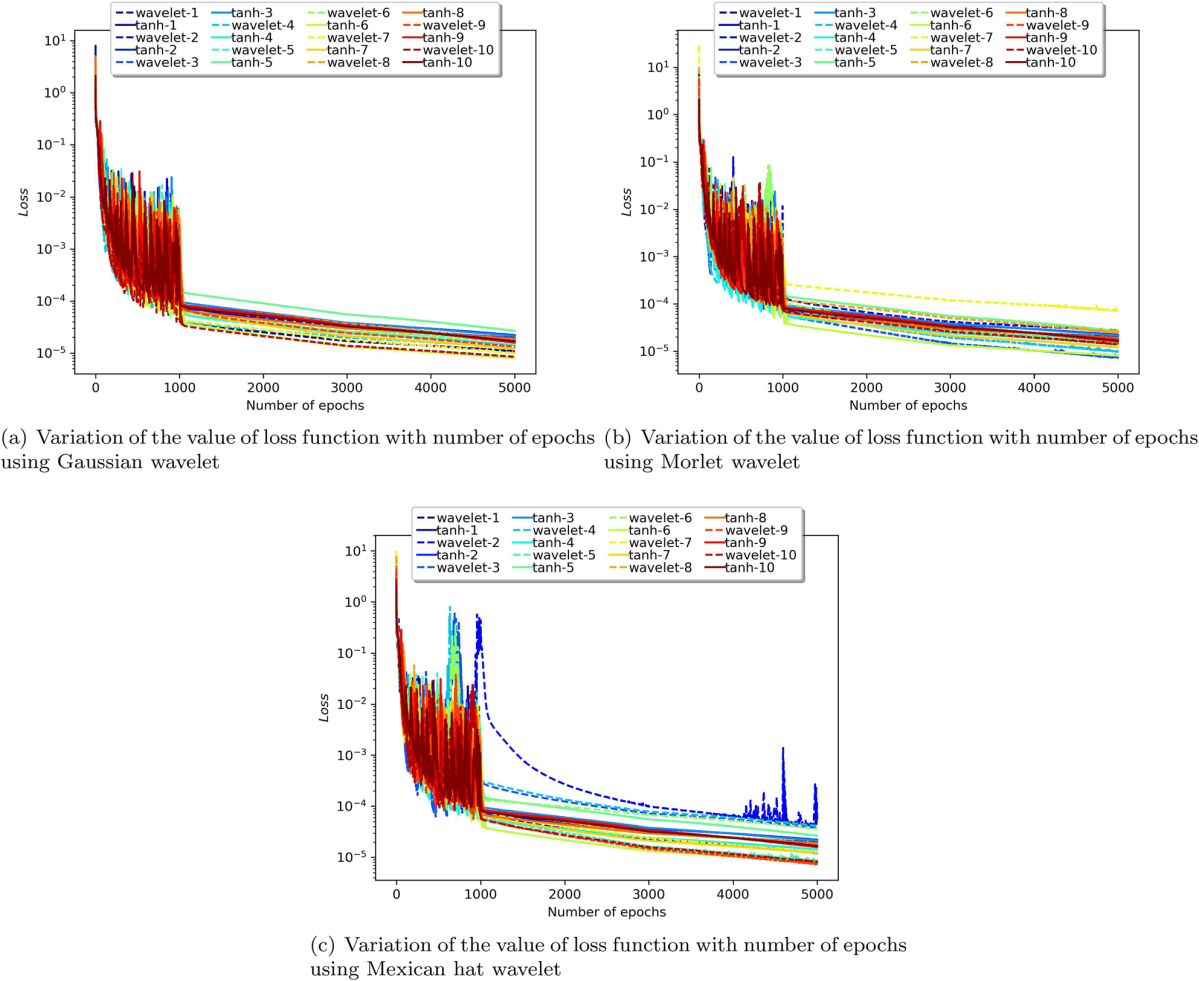
Epoch versus Loss of the proposed method against ‘tanh’ over 10 instances for the Burger’s equation.

The suggested method is extended to solve Burger’s equation for two different cases. In the first case, a model with 20 neurons and 8 hidden layers was utilized to approximate the solution, and effective results were observed. The results were compared with the numerical values of the analytical solution provided in^66^. The absolute error of the obtained solution using the proposed method with that of the analytical solution is tabulated in Table A4 in online Appendix. The solution is illustrated in Fig. 12. The solution using the proposed method against the ‘tanh’ activation function for 6 different values of “*t*” is given in Fig. 13. Figure 14 plots the number of epochs against the loss value for all the considered wavelet activation functions with ‘tanh’ (run over 10 instances) for the considered Burger’s equation. Table 4 provides the average and standard deviation (SD) of relative $L^2$ error of the PINN utilizing various activation functions against the analytical solution for ten different instances. It can be observed that the Gaussian wavelet gives the least error in comparison with the other considered activation functions.

**Table 4:** Average and SD of relative $L^2$ error of the proposed method in solving Burger’s equation (case 1) using different activation function compared against the analytical solution over 10 instances.

Activation	Mean	SD
Gaussian	1.27E−03	8.82E−04
Morlet	1.42E−03	5.14E−04
tanh	2.06E−03	9.30E−04
Mexican hat	2.33E−03	2.55E−03

**Figure 15:**
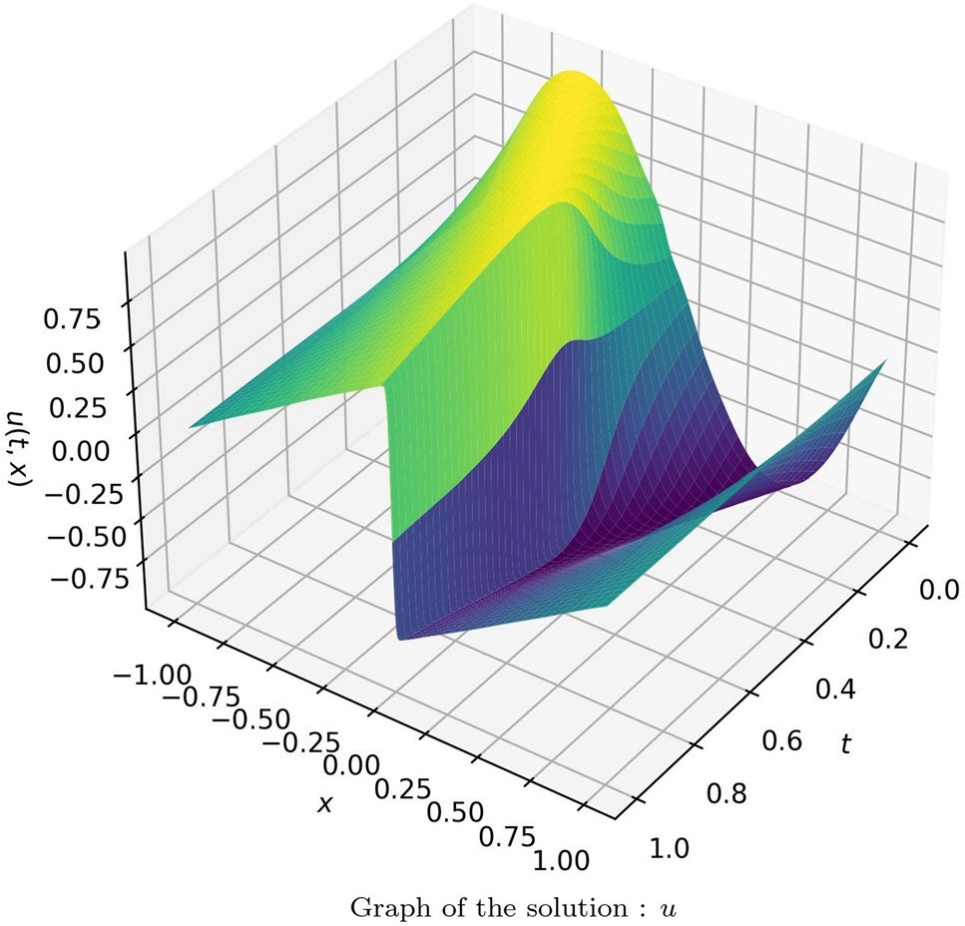
Solution obtained for the Burger’s equation (case 2) using the proposed method with Gaussian wavelet.

**Figure 16:**
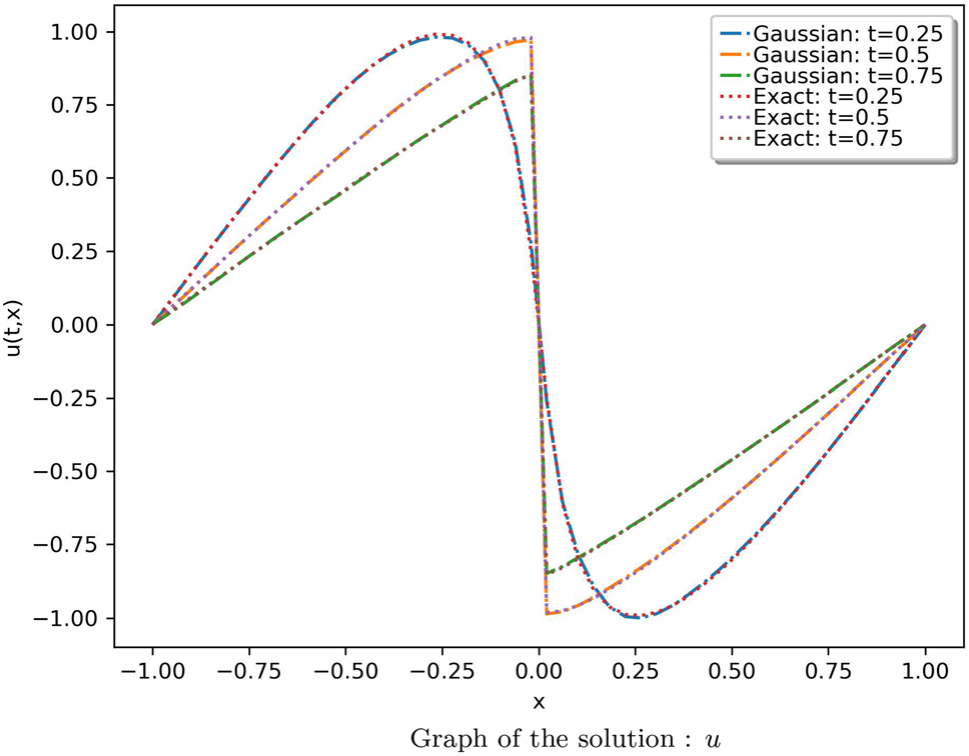
Comparison of the results of proposed method using Gaussian wavelet with the exact solution for different values of t.

In the second case, a model with 20 neurons and 8 hidden layers was utilized to approximate the solution, and effective results were observed. The results were compared with the analytical solution provided in^67^. The absolute error of the obtained solution using the proposed method with that of the analytical solution is tabulated in Table A6 in online Appendix. The solution is illustrated in Fig. 15. The comparison of the solution of the proposed method using Gaussian wavelet with that of the exact solution for 3 different values of “*t*” is given in Fig. 16. Table 5 provides the average and standard deviation (SD) of relative $L^2$ error of the PINN utilizing various activation functions against the analytical solution for ten different instances. It can be observed that the Gaussian wavelet gives the least error in comparison with the other considered activation functions.

**Table 5:** Average and SD of relative $L^2$ error of the proposed method in solving Burger’s equation (case 2) using different activation function compared against the analytical solution over 10 instances.

Activation	Mean	SD
Gaussian	7.50E−03	1.91E−03
tanh	1.02E−02	3.83E−03
Mexican hat	1.65E−02	7.80E−03
Morlet	1.93E−02	9.06E−03

## Conclusion

In this work, the Blasius equation was solved using PINN with wavelet as an activation function, and the method was then extended to solve a linear coupled equation, a non-linear coupled equation and partial differential equations. Three distinct wavelet functions were applied, and the results were compared. The findings of all the problems that were taken into consideration showed that utilizing wavelet as the activation function in PINN produces results with improved accuracy. The impact of a few hyper parameters, such as the number of neurons, hidden layers, and collocation points, were also explored. The effect of using separate neural network models for the solution of coupled equations was also studied and was observed to give the required accuracy with a simpler architecture. The usefulness of the proposed method of using wavelet activation function is validated by comparing the results with the existing results. The absolute and relative $L^2$ errors calculated indicate the effectiveness of the method and its possible applicability to solve more complex problems.

Future study will involve applying the proposed approach to problems that arise in heat and mass transfer-related industrial applications. Additionally, applicability of wavelet activation functions with different machine learning models will be examined for improving the performance.

## Supplementary Information

Supplementary Information.

## Data Availability

All data generated or analysed during this study are included in this submitted manuscript.
