# Supplementary material for: Wavelets based physics informed neural networks to solve non-linear differential equations
Source: Sci Rep. 2023 Feb 18;13:2882. doi: 10.1038/s41598-023-29806-3 (PMC9938906; doi:10.1038/s41598-023-29806-3)
Supplement: Supplementary file 1 — Supplementary Information. [file 41598_2023_29806_MOESM1_ESM.pdf]

Appendix

In this section, we've compiled all of the tabular data for the specific results mentioned in section 4.

x	Gaussian		Mexican hat		Morlet		tanh	
	f	f'	f	f'	f	f'	f	f'
0	6.00E-05	3.00E-04	8.00E-05	1.70E-04	6.50E-04	1.37E-03	8.10E-04	1.90E-04
0.5	3.40E-04	1.88E-03	1.40E-04	9.20E-04	1.27E-03	1.10E-03	6.80E-04	1.22E-03
1	1.67E-03	3.18E-03	8.50E-04	1.91E-03	1.70E-03	7.30E-04	5.00E-04	3.21E-03
1.5	3.61E-03	5.13E-03	2.28E-03	4.32E-03	1.96E-03	9.00E-05	2.41E-03	4.67E-03
2	6.76E-03	6.93E-03	5.01E-03	5.89E-03	1.66E-03	7.70E-04	4.93E-03	4.83E-03
2.5	1.03E-02	7.51E-03	7.91E-03	5.91E-03	1.08E-03	1.96E-03	7.18E-03	4.57E-03
3	1.44E-02	8.55E-03	1.12E-02	7.24E-03	4.10E-04	3.75E-03	9.91E-03	6.55E-03
3.5	1.86E-02	8.22E-03	1.50E-02	7.70E-03	2.31E-03	3.84E-03	1.35E-02	7.41E-03
4	2.27E-02	8.60E-03	1.88E-02	8.13E-03	4.54E-03	5.56E-03	1.72E-02	7.59E-03
4.5	2.71E-02	8.48E-03	2.30E-02	7.82E-03	7.65E-03	5.96E-03	2.10E-02	7.21E-03
5	3.11E-02	8.09E-03	2.67E-02	7.76E-03	1.05E-02	6.25E-03	2.45E-02	7.43E-03
5	8.00E-05	7.00E-05	3.10E-04	4.40E-04	1.50E-04	2.10E-04	1.83E-03	2.10E-04
5.5	7.00E-05	4.10E-04	4.60E-04	5.90E-04	1.50E-04	2.40E-04	1.62E-03	1.70E-04
6	5.10E-04	1.05E-03	1.01E-03	1.38E-03	3.70E-04	4.60E-04	1.67E-03	7.00E-05
6.5	1.02E-03	9.60E-04	1.81E-03	1.72E-03	6.00E-04	3.90E-04	1.69E-03	2.00E-05
7	1.50E-03	1.09E-03	2.60E-03	1.42E-03	7.70E-04	4.00E-04	1.67E-03	4.00E-05
7.5	2.12E-03	1.20E-03	3.28E-03	1.30E-03	1.04E-03	5.50E-04	1.69E-03	9.00E-05
8	2.64E-03	9.40E-04	3.98E-03	1.52E-03	1.28E-03	3.90E-04	1.76E-03	1.60E-04
8.5	3.15E-03	1.09E-03	4.74E-03	1.42E-03	1.47E-03	4.10E-04	1.83E-03	7.00E-05
9	3.66E-03	8.60E-04	5.29E-03	7.50E-04	1.69E-03	4.70E-04	1.83E-03	2.00E-05

Table A 1: The absolute error of the obtained solution and its derivative using the proposed method with that of the WGM for Blasius equation

x	Gaussian		Mexican hat		Morlet		tanh	
	u	v	u	v	u	v	u	v
0	1.09E-02	4.78E-03	8.84E-03	4.58E-03	8.30E-02	3.30E-02	8.29E-02	3.67E-02
0.1	9.83E-03	4.79E-03	7.96E-03	4.57E-03	7.56E-02	3.33E-02	7.49E-02	3.69E-02
0.2	8.82E-03	4.84E-03	7.07E-03	4.54E-03	6.81E-02	3.36E-02	6.69E-02	3.71E-02
0.3	7.78E-03	4.91E-03	6.19E-03	4.52E-03	6.05E-02	3.40E-02	5.90E-02	3.74E-02
0.4	6.70E-03	4.94E-03	5.30E-03	4.50E-03	5.28E-02	3.43E-02	5.09E-02	3.76E-02
0.5	5.60E-03	4.90E-03	4.40E-03	4.45E-03	4.48E-02	3.44E-02	4.26E-02	3.75E-02
0.6	4.50E-03	4.82E-03	3.45E-03	4.35E-03	3.63E-02	3.42E-02	3.40E-02	3.69E-02
0.7	3.35E-03	4.70E-03	2.45E-03	4.24E-03	2.74E-02	3.36E-02	2.51E-02	3.59E-02
0.8	2.11E-03	4.54E-03	1.42E-03	4.09E-03	1.79E-02	3.25E-02	1.58E-02	3.45E-02
0.9	7.90E-04	4.29E-03	3.10E-04	3.86E-03	7.75E-03	3.09E-02	6.08E-03	3.27E-02
1	5.60E-04	3.96E-03	8.50E-04	3.58E-03	3.00E-03	2.86E-02	4.11E-03	3.02E-02

Table A 2: The absolute error of the obtained solution using the proposed method with that of the analytical solution observed for linear coupled equation

x	Gaussian		Mexican hat		Morlet		tanh	
	u	v	u	v	u	v	u	v
0	7.30E-06	1.92E-05	9.68E-05	1.02E-04	3.00E-06	5.40E-06	8.20E-06	1.96E-05
0.1	5.10E-06	1.58E-04	9.31E-05	1.52E-04	4.20E-06	4.51E-05	1.60E-05	9.77E-05
0.2	3.00E-06	8.12E-05	9.47E-05	7.37E-05	9.30E-06	1.92E-05	1.80E-06	5.17E-05
0.3	3.00E-06	1.58E-05	7.92E-05	1.80E-04	6.80E-06	5.08E-05	8.80E-06	1.87E-04
0.4	5.10E-06	1.02E-04	8.24E-05	6.38E-05	2.50E-06	7.10E-06	4.60E-06	1.12E-04
0.5	5.00E-07	1.13E-04	9.85E-05	2.79E-04	1.75E-05	5.61E-05	1.27E-05	9.39E-05
0.6	7.30E-06	7.35E-05	1.01E-04	6.40E-06	4.90E-06	3.43E-05	1.40E-06	1.65E-04
0.7	5.20E-06	4.73E-05	1.02E-04	2.68E-04	6.40E-06	1.98E-05	1.30E-05	1.33E-04
0.8	7.00E-07	2.33E-05	1.20E-04	6.40E-05	6.20E-06	4.10E-06	9.50E-06	6.41E-05
0.9	6.50E-06	1.34E-05	1.32E-04	2.30E-04	7.20E-06	6.72E-05	2.50E-06	1.05E-04
1	6.00E-06	8.10E-06	1.46E-04	1.31E-04	7.30E-06	1.66E-05	5.40E-06	1.20E-06

Table A 3: The absolute error of the obtained solution using the proposed method with that of the analytical solution observed for non-linear coupled equation

x	t	Gaussian	Mexican hat	Morlet	tanh
0.1	0.5	6.25E-04	1.52E-03	1.16E-03	4.70E-05
0.2	0.5	2.83E-04	1.46E-03	6.51E-04	5.20E-05
0.3	0.5	1.60E-04	1.14E-03	9.10E-05	2.76E-04
0.4	0.5	3.77E-04	6.80E-04	2.32E-04	2.87E-04
0.5	0.5	4.14E-04	5.61E-04	3.26E-04	1.71E-04
0.6	0.5	2.21E-04	6.37E-04	1.60E-05	4.40E-05
0.7	0.5	1.48E-04	1.05E-03	5.10E-05	1.63E-04
0.8	0.5	1.47E-04	7.92E-04	5.10E-05	1.46E-03
0.9	0.5	2.11E-04	4.26E-04	9.90E-05	3.82E-03
0.1	1	1.85E-04	3.94E-04	9.00E-05	1.34E-04
0.2	1	7.60E-05	6.57E-04	2.49E-04	1.97E-04
0.3	1	1.55E-04	1.14E-04	3.65E-04	1.96E-04
0.4	1	9.00E-05	2.93E-04	7.00E-05	2.21E-04
0.5	1	4.70E-05	4.96E-04	3.20E-05	2.25E-04
0.6	1	8.50E-05	4.51E-04	1.92E-04	3.72E-04
0.7	1	3.38E-04	1.93E-04	7.50E-05	6.03E-04
0.8	1	3.69E-04	5.49E-04	4.10E-05	2.65E-04
0.9	1	3.95E-04	2.82E-03	8.90E-05	1.53E-03

Table A 4: The absolute error of the obtained solution using the proposed method with that of the analytical solution for Burger's equation (case 1)

Hidden layers	Neurons	Relative L^2 error	
		u	v
4	4	3.68E-01	5.17E-01
4	10	9.05E-02	1.30E-01
4	24	8.03E-02	1.16E-01
5	10	9.32E-02	1.34E-01
5	16	4.99E-02	7.06E-02
5	20	9.62E-02	1.38E-01
9	10	7.35E-02	1.06E-01
9	20	3.68E-02	5.34E-02
10	20	6.34E-02	9.22E-02

Table A 5: The relative L^2 error in the case of using single model for few different neurons and hidden layers for linear coupled equation

x	t	Gaussian	Mexican hat	Morlet	tanh
-1	0.25	2.86E-03	1.99E-03	8.84E-04	2.25E-03
-0.6	0.25	4.46E-03	5.08E-04	1.44E-03	2.14E-03
-0.2	0.25	5.89E-02	4.96E-02	7.78E-03	4.26E-02
0.2	0.25	2.70E-02	2.29E-02	3.73E-03	1.02E-02
0.6	0.25	7.03E-03	6.49E-03	3.37E-03	1.50E-03
1	0.25	1.48E-03	4.00E-04	9.48E-04	1.78E-03
-1	0.5	3.12E-03	3.00E-04	9.51E-04	9.15E-04
-0.6	0.5	2.43E-03	1.52E-03	1.76E-03	3.35E-03
-0.2	0.5	1.38E-02	1.00E-02	2.30E-03	7.28E-03
0.2	0.5	2.12E-02	1.33E-02	5.02E-03	2.17E-03
0.6	0.5	4.00E-03	8.98E-04	4.63E-04	2.06E-03
1	0.5	2.26E-03	1.42E-03	9.22E-04	3.13E-03
-1	0.75	8.89E-04	1.92E-03	1.11E-03	3.35E-04
-0.6	0.75	1.52E-03	6.89E-05	1.97E-04	1.24E-03
-0.2	0.75	4.43E-03	3.08E-03	2.41E-03	2.29E-03
0.2	0.75	2.48E-03	4.60E-03	2.13E-03	3.34E-04
0.6	0.75	1.87E-03	3.00E-03	3.41E-04	2.05E-03
1	0.75	1.35E-03	3.31E-03	1.10E-04	4.14E-03

Table A 6: The absolute error of the obtained solution using the proposed method with that of the analytical solution for Burger's equation (case 2)
